# *Coccomyxa antarctica* sp. nov. from the Antarctic lichen *Usnea aurantiacoatra*

**DOI:** 10.3897/phytokeys.98.25360

**Published:** 2018-05-16

**Authors:** Shunan Cao, Fang Zhang, Hongyuan Zheng, Chuanpeng Liu, Fang Peng, Qiming Zhou

**Affiliations:** 1 Key Laboratory for Polar Science SOA, Polar Research Institute of China, No.451 Jinqiao Road, Pudong Avenue, Shanghai, 200136, China; 2 College of Environmental Science and Engineering, Tongji University, Shanghai 200092, China; 3 School of Life Science and Technology, Harbin Institute of Technology, 2 Yikuang Street, Nangang Distinct, Harbin, 150080, China; 4 China Centre for Type Culture Collection (CCTCC), College of Life Sciences, Wuhan University, No. 299 Bayi Road, Wuchang District, Wuhan 430072, China

**Keywords:** Lichen epiphyte, morphology, TEM, phylogeny

## Abstract

The single celled green alga *Coccomyxa antarctica* Shunan Cao & Qiming Zhou, **sp. nov.** was isolated from the Antarctic torrential lichen *Usnea aurantiacoatra* (Jacq.) Bory. It is described and illustrated based on a comprehensive study of its morphology, ultrastructure, ecology and phylogeny. *C. antarctica* is a lichenicolous alga which has elongated cells and contains a parietal chloroplast as observed under the microscope. *C. antarctica* is clearly different from other species by phylogenetic analysis (ITS rDNA and SSU rDNA sequences), also it differs from its phylogenetic closely species *C. viridis* by its larger cell size.

## Introduction

Lichens, the typical symbiosis, generally consist of one fungal partner and its photosynthetic partner alga (usually a green alga or a cyanobacterium). With the development of research techniques, many other eukaryotic (Wilkinson et al. 2015, Spribille et al. 2016) and prokaryotic microbes (Aschenbrenner et al. 2016) have been observed in concurrence with lichen thalli besides the mycobiont and photobiont partners, such as lichenicolous fungi (Edwards et al. 2017, Asplund et al. 2017) and algae (Gustavs et al. 2017).

The green algae of the genus *Coccomyxa* (Trebouxiophyceae, Chlorophyta) are distributed worldwide and can be found in both aquatic and terrestrial habitats, in free living and symbiotic status (Malavasi et al. 2016). The species of *Coccomyxa* can be lichenicolous algae or lichenised photosynthetic partners in lichens (Malavasi et al. 2016). Historically, the taxonomy of this genus has been problematic. Originally a total of 14 free living species, 13 lichenised species and six lichen epiphytic species were summarised by Jagg (1933) based on morphology. Recently, a total of seven species has been distinguished, since the morphological characters of the unicellular green algae *Coccomyxa* vary in different environments and a DNA-based identification approach was proposed by Darienko et al. (2015). Subsequently, an improved method based on phylogenetic and ecological features was used for delimiting the species of this genus and 27 species scenario were recognised (Malavasi et al. 2016). The combination of ecological and DNA sequences data seems to be effective in distinguishing the *Coccomyxa* species.

In this current study, an epiphytic green alga was isolated from the Antarctic lichen *Usnea aurantiacoatra* (Jacq.) Bory. It will be demonstrated that this green alga is new to science based on the comprehensive analysis approach including morphology, ultrastructure, ecology and phylogeny.

## Methods

### Isolation and culture

During the 30^th^ Chinese National Antarctic Research Expedition (from 1^st^ Feb. 2014 to 15^th^ March 2014), a specimen of Antarctic lichen *U. aurantiacoatra* was collected from Fildes Peninsula, King George Island, (62°12.70'S, 58°55.70'W). The specimen was incubated at 4 °C till the isolation was processed.

An *Usnea aurantiacoatra* specimen (d-B1), kept in the Resource-sharing Platform of Polar Samples which includes samples of Biology, Ice-snow, Rock, Deep-space and Sediment (BIRDS ID 2131C0001ASBM100063), was used to isolate the green alga. One green alga (Ua6) (Freshwater Algae Culture Collection at the Institute of Hydrobiology, FACHB-2140) was isolated by an improved tissue culture procedure: 1. Washing lichen tissues (2–3 pieces, about 5 mm of each) three times in sterile water; 2. Grinding each piece of tissue in a 1.5 ml centrifuge tube by a mini glass pestle; 3. Sifting the fragments through three different screen meshes (hole sizes: 0.35 mm, 0.10 mm and 0.03 mm); 4. Washing the fragments in the mesh whose hole size was 0.03 mm for 5 min with sterile water, repeating three times; 5. Selecting the fragments on the 0.03 mm-mesh (the size of these fragments is between 0.03 mm and 0.10 mm) and then culturing them on PDA and BBM petri-dish medium. All the operations were undertaken under aseptic conditions. The isolations were incubated in an illumination incubator (4 °C, 12 hr light/12 hr dark, natural lighting). The algal cultures were maintained in both PDA and BBM petri-dish medium at 4 °C.

### Microscope and transmission electron microscopy (TEM) analysis

Compound microscopes (Nikon Eclipse 80i and Nikon ACT-1 V2.70) were used for morphology observation and photographing the algal cultures.

For transmission electron microscopy (TEM) observation, algal cells were fixed with 2.5% glutaraldehyde in phosphate buffer (0.1 M, pH 7.4) for 2 h, washed using the same buffer for 15 min and repeated three times, then post-fixed using 1% OsO_4_ fixing solution for 3 h and washed using the same phosphate buffer for 15 min, three times. Samples were dehydrated in a graded ethanol series and replaced by propylene oxide. All the procedures above were operated at 4 °C. The samples were embedded using Spurr resin kit (Spi-Chem, USA). The resin was polymerised at 37 °C overnight, 45 °C for 12 h and 60 °C for 48 h. Thin sections (70 nm) were cut with a Leica EM UC6 (Germany) and stained with 3% uranyl acetate and lead citrate. The collections were observed using a JEM1230 (JEOL, Japan) electron microscope at 80–120kV. Micrographs were acquired by an Olympus SIS VELETA CCD camera equipped with iTEM software.

### Molecular analysis

Genomic DNA of the green alga was extracted by a modified CTAB method (Cao et al. 2015a). The SSU rDNA was amplified using eukaryote universal primer pairs NS1, NS4; NS3, NS6; NS5, NS8 (White et al. 1990). The ITS rDNA was amplified by the primer pair ITS5, O2 (Cao et al. 2015a). A total volume of 50 µl PCR reaction was selected, the PCR application conditions and products verification following Cao et al. 2015a. Double-stranded PCR products were sequenced with an ABI 3730XL sequencer.

Double-directional sequences data of ITS nrDNA and SSU nrDNA were checked and assembled using the SEQMAN programme within the Lasergene v7.1 software package (DNASTAR Inc.), respectively. The regions of rDNA flanking the ITS region were trimmed off. Preliminary alignment of the sequences obtained in the present study and those retrieved from GenBank (Table 1) was performed using the ClustalW algorithm included in MEGA 5 and then adjusted manually (Tamura et al. 2011). The phylogenetic structure of each alignment was constructed using a Neighbour Joining (NJ) method. The reliability of the inferred trees was tested using bootstrap searches of 1000 resamplings. Altogether, 35 ITS nrDNA and 37 SSU nrDNA sequences, used in the phylogenetic analysis, were retrieved from GenBank (Table 1). The sequence representing the new species was sequenced by the authors and submitted to GenBank (MF465900).

**Table 1.** Sequences used in the present study.

Species	Collection No.	GenBank No.	
		ITS rDNA	SSU rDNA
Clade B*Coccomyxa sp.	GA5a	AB917140	AB917140
Clade C* *Chlorella saccharophila*	CCAP 211/60		FR865679
Clade D* *Coccomyxa* sp.	CCAP 216/24	FN298927	FN298927
	CCAP 812/2A	HG972992	HG972992
Clade E* *Coccomyxa* sp.	IB-GF-12		KM020052
Clade E* *Coccomyxa subellipsoidea*	CCAP 812/3	HG972972	HG972972
Clade H* *Coccomyxa* sp.	KN-2011-U5	HE586557	
Clade I* *Coccomyxa* sp.	KN-2011-T3	HE586515	HE586515
	KN-2011-T1	HE586550	
Clade J* *Pseudococcomyxa simplex*	CAUP H 103		HE586505
Clade K* *Coccomyxa* sp.	KN-2011-C4	HE586508	HE586508
Clade L* *Monodus* sp.	UTEX B SNO83		HE586506
Clade M* *Monodus* sp.	CR2-4	HE586519	HE586519
Clade N* *Coccomyxa viridis* 3	CAUP H5103	HG973007	HG973007
	SAG 2040	HG973004	HG973004
*Coccomyxa actinabiotis*	216-25	FR850476	FR850476
*Coccomyxa actinabiotis*	KN-2011-T4	HE586516	HE586516
***Coccomyxa antarctica***	**Ua6 (FACHB-2140)**	**MF465900**	**MF465900**
*Coccomyxa avernensis*	SAG 216-1		HG972999
*Coccomyxa avernensis*	Wien C19	HG973000	HG973000
*Coccomyxa dispar*	SAG 49.84	HG972998	HG972998
*Coccomyxa elongata*	CAUP H5107	HG972981	HG972981
	SAG 216-3b	HG972980	HG972980
*Coccomyxa galuniae*	CCAP 211/97	FN298928	FN298928
	SAG 2253	HG972996	HG972996
*Coccomyxa melkonianii*	SCCA048	KU696488	KU696488
*Coccomyxa onubensis*	ACCV1	HE617183	HE617183
*Coccomyxa polymorpha*	CAUP H5101	HG972979	HG972979
	KN-2011-T2	HE586514	HE586514
*Coccomyxa simplex*	CAUP H 102	HE586504	HE586504
	SAG 216-2	HG972989	HG972989
*Coccomyxa solorinae*	SAG 216-12	HG972987	HG972987
	SAG 216-6	HG972988	HG972988
*Coccomyxa subellipsoidea*	SAG 216-7	HG972976	HG972976
	Wien C20	HG972975	HG972975
	CAUP H5105	HG972974	
*Coccomyxa vinatzeri*	ASIB V16	HG972994	HG972994
*Coccomyxa viridis*	SAG 216-14	HG973002	HG973002
	SAG 216-4	HG973001	HG973001
*Elliptochloris bilobata*	SAG 245.80	HG972969	HG972969
*Hemichloris antarctica*	SAG 62.90	HG972970	HG972970

Note: * Clades referred to Malavasi et al. (2016); The information about the new species *Coccomyxa antarctica* is marked in bold.

## Results

**Figure 1.**
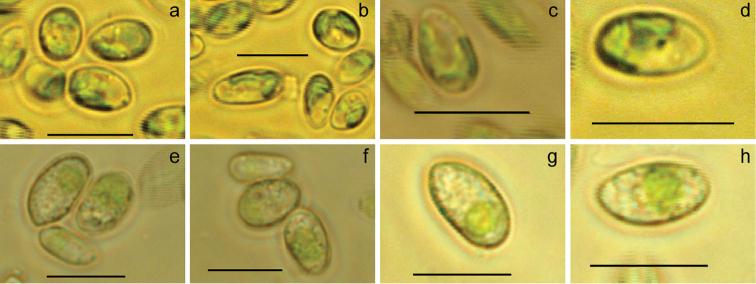
Morphology of *Coccomyxa antarctica* Shunan Cao & Qiming Zhou, sp. nov. **a–d** cultured in BBM medium; e-f cultured in PDA medium. Scale bars: 10 μm.

**Figure 2.**
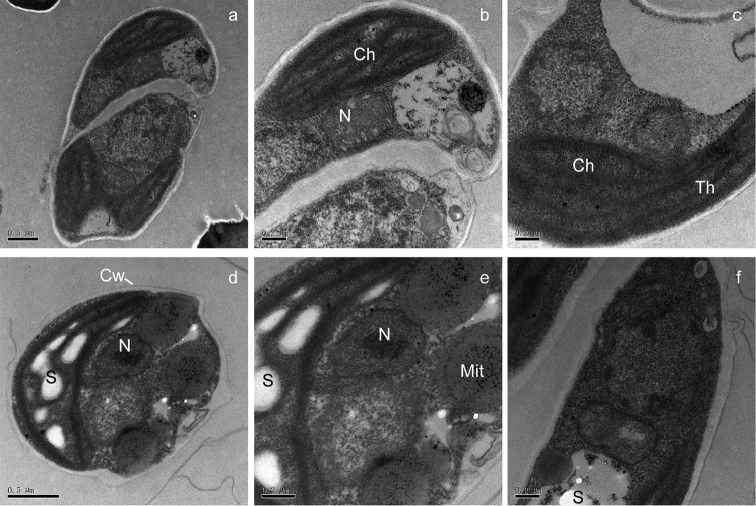
Ultrastructure of *Coccomyxa antarctica* Shunan Cao & Qiming Zhou, sp. nov. **a–c** cultured in BBM medium; d-f cultured in PDA medium. **a, b** mature autosporangium **c, d** Cup-shaped chloroplast **e, f** vegetative cell. Key: Ch: chloroplast; Cw: cell wall; Mit: mitochondria; N: nucleus; S: starch granules; Th: thylakoids. Scale bars: 0.5 μm(**a, d**); 0.2 μm (**b, c, e, f**).

We examined the algal strain (Ua6) isolated from Antarctic lichen *Usnea aurantiacoatra* using both morphological identification and molecular markers. The isolated alga Ua6 was observed with elongated cells (4–7 µm wide and 8–12 µm long), whose cell wall was thin and smooth, each cell contained a parietal chloroplast (Figure 1); no pyrenoid was observed within their chloroplast using transmission electron microscopy (Figure 2). The alga strain Ua6 appeared to have a shorter growth cycle when cultured in PDA medium than that in BBM medium, but no significant morphological differences were observed from the cells cultured in PDA and BBM mediums (Figure 1).

The phylogenetic analysis of both ITS rDNA and SSU rDNA supported that the isolated green alga Ua6 was an undescribed *Coccomyxa* species. For the ITS rDNA, the sequences of *Coccomyxa* clustered as six subgroups. The newly isolated green alga Ua6, *C. viridis*, *C. avernensis*, *Coccomyxa* sp. Clade M, Clade N and Clade KL clustered as a subgroup, was supported with a bootstrap value 100, but the new species Ua6 was clearly different from the other species in this subgroup according to the branch length. For the SSU rDNA, the sequences of *Coccomyxa* clustered as five subgroups. The newly isolated green alga Ua6 also showed a close relationship with *C. viridis*, *C. avernensis*, *Coccomyxa* sp. Clade K, Clade L, Clade M and Clade N as a well-supported subgroup with the bootstrap value 100. Furthermore, the SSU rDNA sequence of Ua6 was clearly distinguished from the other species.

According to the comprehensive study of both morphological and phylogenetic analysis, the isolated single cell green algae Ua6 is a newly reported species and here described as new:

### Coccomyxa antarctica

Taxon classificationPlantaeBivalvulidaMyxidiidae

Shunan Cao & Qiming Zhou sp. nov.

Figures 1 , 2

#### Holotype.

Preparation FACHB-2140, Freshwater Algae Culture Collection, the Institute of Hydrobiology (FACHB-Collection) represented here by Figure 1d.

#### Type locality.

Antarctic, Fildes Peninsula, on stone (62°12.70'S, 58°55.70'W), 44 m a.s.l., Isolated from the Antarctic lichen *Usnea aurantiacoatra* (d-B1, BIRDS ID: 2131C0001ASBM100063) on 5^th^ May 2014.

#### Diagnosis.

**Figure 3.**
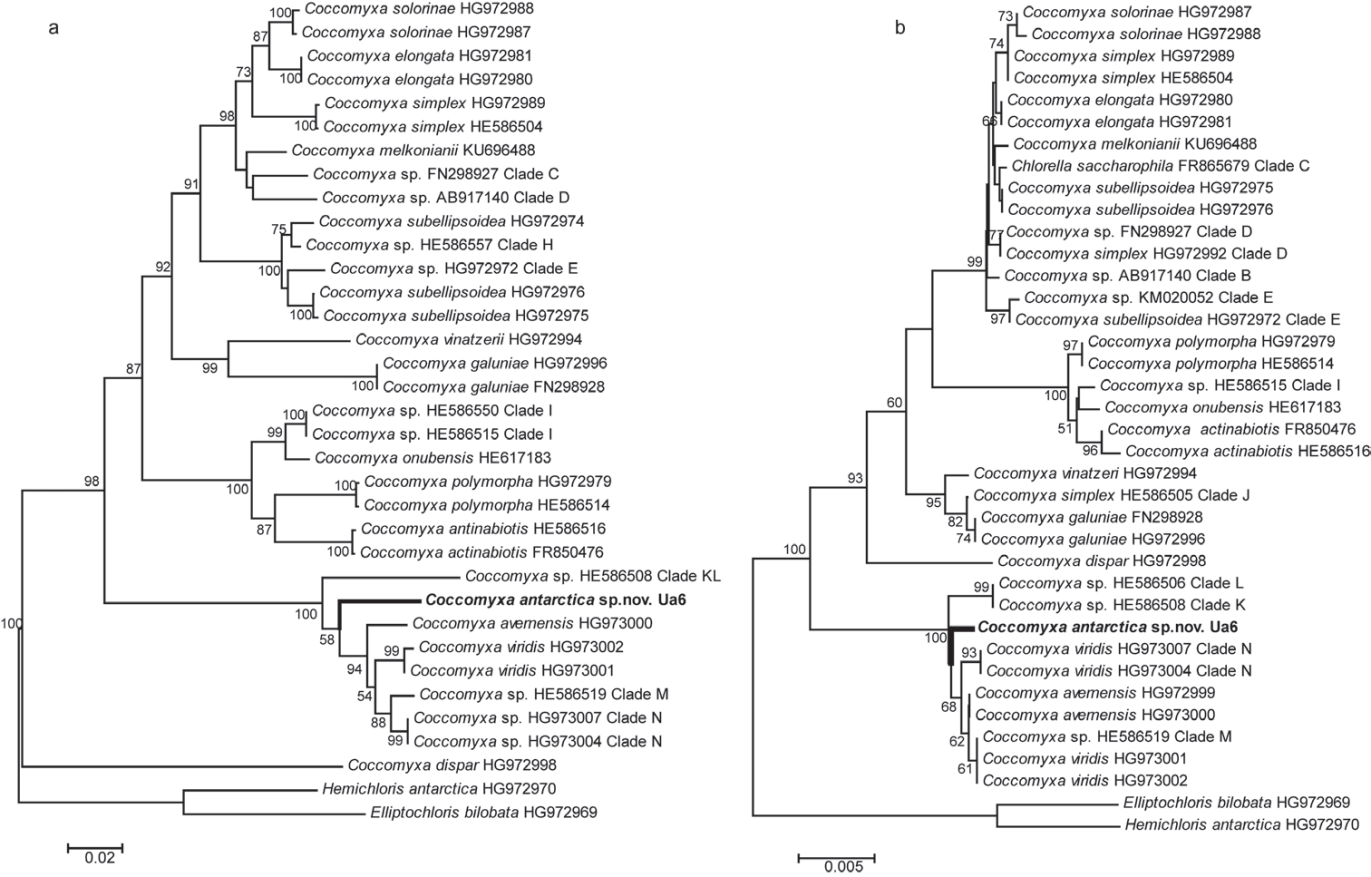
The NJ tree based on ITS rDNA (**a**) and SSU rDNA (**b**) sequences phylogenetic analyses. The sequences obtained by the authors were exhibited in bold font. The clades referred to Malavasi et al. (2016).

The vegetative cells are ovoid to ellipsoidal, asymmetrical, 4–7 µm wide and 8–12 µm long; some cells were sub-sphaeroidal in BBM medium, without mucilaginous sheath. Cell wall smooth, double in ultrastructures. Protoplast with single central cell nucleus, filled with lipid droplets. Chloroplast parietal, with starch granules in interthylacoidal spaces, without pyrenoid. Reproductive cells were not observed. It looks morphologically similar to other *Coccomyxa* species but differs from other species of *Coccomyxa* in ITS rDNA (Table 1 & Figure 3a) and SSU rDNA (Table 1, Figure 3b).

#### Habitat.

Epiphytic green alga, living with lichen *Usnea aurantiacoatra* in harsh environments (Antarctic).

## Discussion

The morphological and ultrastructure characters indicate that the isolated green alga Ua6 is a *Coccomyxa* species, which is characterised by ovoid to ellipsoidal single cells. The isolated strain Ua6 is morphologically similar to the other *Coccomyxa* species, but different from the phylogenetic closely related species *C. viridis* by its larger cell size (4–7 µm wide and 8–12 µm long vs 1.8–3.6 µm wide and 4.7–8.4 µm long) (Hodač 2015). However, the morphological characters are not stable and non-credible as they change under different environments or culture conditions. For example, the cell shape is significantly dependent on culture conditions (Tsarenko and John 2011) and the mucilaginous sheaths are highly dependent on nutrient availability which is the key trait in separating *Coccomyxa* and *Pseudococcomyxa* (Darienko et al. 2015).

Since the molecular barcode provides a more stable and informative tool in identification and classification of the species of *Coccomyxa* (Darienko et al. 2015, Malavasi et al. 2016), both the ITS rDNA and SSU rDNA phylogenetic analyses were applied in the current study. The results supported the observation that the single cell green alga *Coccomyxa antarctica* sp. nov. is different from the other reported species of *Coccomyxa*, indicating that it is a species new to science.

Furthermore, species of *Coccomyxa* have been reported as photobionts of lichen genera *Baeomyces*, *Dibaeis*, *Icmadophila*, *Lichenomphalia*, *Micarea*, *Multiclavula*, *Nephroma*, *Orceolina*, *Peltigera*, *Placynthiella*, and *Solorina* in earlier studies (Poulsen et al. 2001, Smith et al. 2009, Wirth et al. 2013, Gustavs et al. 2017), but not *Usnea*. The authors’ earlier studies also revealed that the photosynthetic partner of the Antarctic lichen *U. aurantiacoatra* was *Trebouxia jamesii* (Hildreth and Ahmadjian) Gärtner (Cao et al. 2015b, Cao et al. 2017); as a result, the isolated green alga *Coccomyxa antarctica* sp. nov. is not lichenised alga, but a lichen epiphytic alga.

## Supplementary Material

XML Treatment for Coccomyxa antarctica
